# Diagnostic and Therapeutic Roles of the “Omics” in Hypoxic–Ischemic Encephalopathy in Neonates

**DOI:** 10.3390/bioengineering9100498

**Published:** 2022-09-22

**Authors:** Girish Kumar Rasineni, Nalinikanta Panigrahy, Subha Narayan Rath, Madhurarekha Chinnaboina, Ramesh Konanki, Dinesh Kumar Chirla, Srinivas Madduri

**Affiliations:** 1 LCMS Division, Tenet Medcorp Pvt. Ltd., 54 Kineta Towers Road No 3, Banjara Hills, Hyderabad 500034, India; 2 Department of Neonatology, Rainbow Children’s Hospital, Hyderabad 500034, India; 3 Regenerative Medicine and Stem Cell Laboratory, Department of Biomedical Engineering, Indian Institute of Technology Hyderabad, Telangana 502284, India; 4 Department of Pediatric Neurology, Rainbow Children’s Hospital, Hyderabad 500034, India; 5 Bioengineering and Regenerative Medicine, Department of Biomedical Engineering, University of Basel, University Hospital Basel, 4001 Basel, Switzerland; 6 Department of Surgery, Bioengineering and Neuroregeneration, University of Geneva, University Hospital Geneva, 1211 Geneva, Switzerland

**Keywords:** hypoxic–ischemic encephalopathy, genomics, proteomics, metabolomics, biomarkers, omics

## Abstract

Perinatal asphyxia and neonatal encephalopathy remain major causes of neonatal mortality, despite the improved availability of diagnostic and therapeutic tools, contributing to neurological and intellectual disabilities worldwide. An approach using a combination of clinical data, neuroimaging, and biochemical parameters is the current strategy towards the improved diagnosis and prognosis of the outcome in neonatal hypoxic–ischemic encephalopathy (HIE) using bioengineering methods. Traditional biomarkers are of little use in this multifactorial and variable phenotype-presenting clinical condition. Novel systems of biology-based “omics” approaches (genomics, transcriptome proteomics, and metabolomics) may help to identify biomarkers associated with brain and other tissue injuries, predicting the disease severity in HIE. Biomarker studies using omics technologies will likely be a key feature of future neuroprotective treatment methods and will help to assess the successful treatment and long-term efficacy of the intervention. This article reviews the roles of different omics as biomarkers of HIE and outlines the existing knowledge of our current understanding of the clinical use of different omics molecules as novel neonatal brain injury biomarkers, which may lead to improved interventions related to the diagnostic and therapeutic aspects of HIE.

## 1. Introduction and Pathophysiology of HIE

Hypoxic–ischemic encephalopathy (HIE) is a complex disease process caused by the disruption of the cerebral blood flow and a subsequent hypoxic event in the affected area of the brain, leading to the risk of short- and long-term neurological disability and death [1,2]. Perinatal asphyxia leading to neonatal encephalopathy (NE) is the most common cause of death and neurodisability in term infants, with an incidence range of 10 to 20 per 1000 live births in low- and middle-income countries, though less cases occur in high-income countries [3,4].

Hypoxic–ischemic brain injury affects the fetal brain less severely than the adult brain. This is because the fetal brain receives a constant ATP supply by metabolizing lactate, ketones, glucose, and other molecular substrates. Hence, it always has an energy reserve, which helps in sustaining hypoxia–ischemia. However, in severe cases of ATP deficit, the fetal brain still can be injured. Conditions such as preeclampsia, umbilical cord knotting, umbilical cord prolapse, chronic maternal hypoxia, shoulder dystocia, and placental rupture can hinder the oxygenated cerebral blood flow and lead to changes in cellular and systemic responses [5,6].

**Figure 1:**
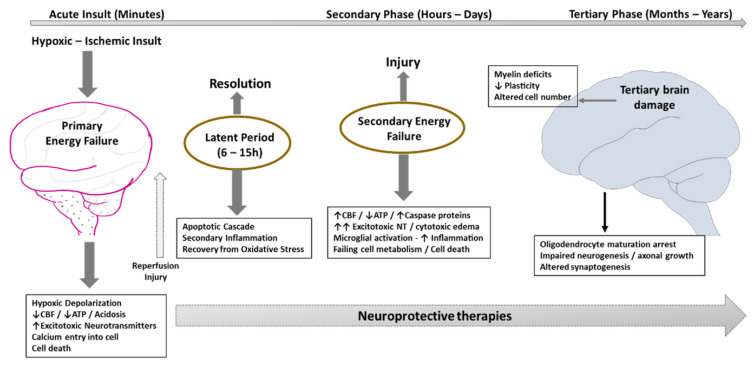
The three phases of development of the pathophysiology of hypoxic–ischemic encephalopathy (HIE).

Hypoxic–ischemic brain injury progresses gradually over time and can be distinguished into phases (Figure 1). The inflammatory response begins when there is a critical energy failure. This initiates an uncontrolled excitatory neurotransmitter release, which further starts an ischemic cascade that leads to neuronal damage at the mitochondrial and cytoplasmic levels, and then the blood–brain barrier disruption occurs. The increasing amount of membrane peroxidation also increases the rigorousness of the ATP depletion, which activates important inflammatory responses [7].

First, in the acute phase, the primary energy failure occurs in minutes. This leads to oxidative metabolism changes, cytotoxic edema, and the accumulation of excitotoxins. Subsequently, in the secondary phase, there is a restoration of the post-cerebral circulation, leading to the beginning of a latent phase (lasting for about 6–15 h). The tertiary phase, which starts after 6–15 h, causes a secondary energy failure that can last for days to months.

**Figure 2:**
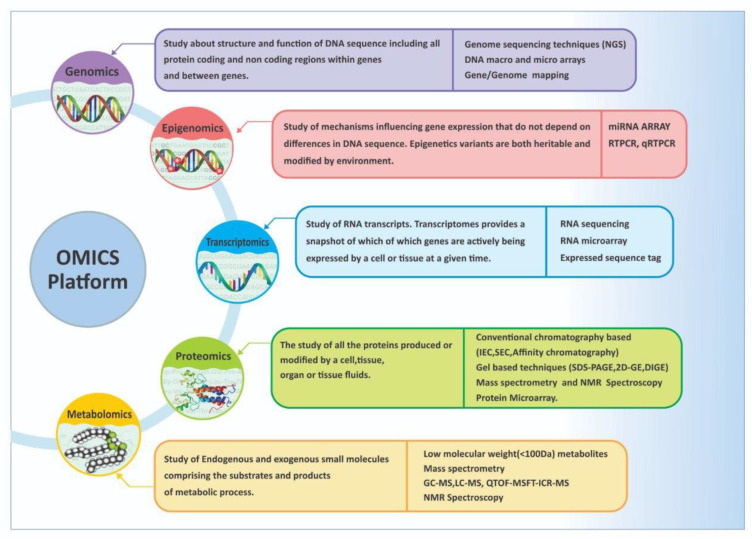
Principles of omics in clinical practice.

There are five major events occurring in HIE, which include (a) mitochondrial dysfunction, (b) oxidative stress, (c) intracellular Ca^2+^ accumulation, (d) excitotoxicity, and € inflammation [8,9]. The current diagnostic techniques for HIE include the findings from a number of investigations, such as clinical Apgar scores, umbilical cord blood investigations, neonatal blood levels of lactate and base excess, liver function and renal function tests, and neuroimaging [10]. Neuroimaging is used for better prediction at 3 to 10 days of life, and not immediately at birth. Therefore, the current diagnostic methods fail to predict the severity of HIE and their positive predictive ability is low. Although therapeutic hypothermia started before 6 h until 72 h can be lifesaving in these neonates, there is no test to confirm HIE in the majority of cases [11]. All of the standard biochemical parameters are poor predictors for the HIE diagnosis, severity classification, and prognosis [10]. However, it is unconvincing that a single diagnostic approach will reflect the full spectrum of the injured brain in any multifactorial disease such as HIE [12]. The major challenges are as follows: (1) our inability to differentiate between a difficult perinatal transition without encephalopathy from mild HIE; (2) the identification of susceptible neonates who might progress to severe HIE [13]; (3) our inability to predict the response to therapeutic hypothermia and the possible need for other neuroprotective interventions; (4) the need to find the long-term outcomes related to the severity of the disease and their management approaches. A focus on specific genes, proteins, or metabolites in isolation may not provide deeper insights into the stage of the disease or the disease severity. It has been suggested that a “multi-omics” approach (Figure 2) might be more effective. As currently a number of bioengineering methods have been developed to detect the many biosensors, these methods might be useful in predicting the diagnostic and therapeutic outputs of HIE in future.

Omics technologies are high-throughput techniques that gather specific data about a clinical condition or disease, such as genes from the genome, proteins from tissue, or metabolites. The examples of these technologies include next-generation sequencing, used to study genomics and transcriptomics, and mass spectrometry (MS), used to study proteomics and metabolomics [14]. Omics technologies for precision diagnoses are not direct, but rather work via the identification of important biomarkers.

## 2. Genomics

The primary DNA sequence remains unaltered throughout the lifespan in the absence of somatic mutations. Genomics focuses on discovering genetic variants associated with the disease, the response to treatment, or the prognosis. The initial hypoxia and subsequent oxidative stress start a cascade of biochemical processes at the cellular level, resulting in brain injury [15]. The microglia respond vigorously during HIE changes and produce excess inflammatory cytokines, in association with glutamate, nitric oxide (NO), and reactive oxygen species (ROS) [16]. The production of NO in the endothelial cells of blood vessels is regulated by the NOS3 (nitric oxide synthase 3) gene, and polymorphisms of this gene were found to alter the NO synthase function associated with HIE [17]. Polymorphisms located in the promoter region, upstream of the NOS3 gene, might influence the transcription and reduce the gene expression, leading to a reduced production of NO [18]. The different biomarkers at the genomics level are summarized in Table 1.

Similarly, polymorphisms and mutations in the angiotensinogen gene of the renin–angiotensin system may increase the level of angiotensin II [28], which was found to give rise to the contraction, hyperplasia, hypertrophy, and lipid deposition of arteriolar vascular smooth muscles, thereby causing the hardening of smaller vessels and resulting in ischemic changes in subcortical and deep white matter [20,29]. Mouse model studies showed that nicotine exposure reduces angiotensin II type 2 receptor expression in the developing brain, increasing the vulnerability to HIE injury in neonates [22]. Angiotensin-II-induced neuronal apoptosis can also be observed through the mTOR signaling pathway after HIE [30]. However, a single marker approach might not explain the genetic reasons for a complex condition such as HIE, and an integrated genomic analysis needs to be conducted to identify markers leading to brain injury. The identification of novel neuronal target molecules acting in regulating the onset of the delayed events in cell death has the potential to prevent the severity of the neuropathological effects of perinatal brain injury.

## 3. Transcriptomics

Transcriptomics provides insights of the regulation of our genes and their expression in different biological tissues and events. Genes exert their biological effects via transcription to messenger RNA in any tissue. Unlike the DNA sequence, which is mostly fixed in a person, there is a high fluctuation of the transcription in different tissues and in response to physiological and pathological stimuli [31,32]. Technical advances can at present be used to find a quantitative measurement of all expressed genes (the ‘transcriptome’) in any tissue at a particular time, which helps in depicting complex biological systems and in developing biomarkers specific to the system [33]. A number of biomarkers at the transcriptomic level are summarized in Table 1. MicroRNAs (miRNAs) are evolution-wise preserved short noncoding RNA molecules containing approximately 20–25 nucleotides that cause RNA silencing and the post-transcriptional regulation of gene expression [34]. The use of miRNA-guided diagnostics is a novel molecular approach to depicting clinically significant information from patient samples [35]. In newborns with HIE, miRNAs as biomarkers will help to better understand the state of the disease. Circulating extracellular miRNAs (exmiRNAs) in the blood have been implicated in cell–cell communication. Therefore, they may provide useful biological information about the inter-relationships among the maternal, fetal, and placental genes expressed during pregnancy [36].

The circulating miRNAs, which are regulated by hypoxia, can be measured in the maternal blood. This can lead to the identification of biomarkers in utero [27]. A recent study demonstrated that a panel of hypoxia-induced miRNAs could be detected in the maternal blood, and that their expression changed when the fetus was subjected to hypoxia, either in the acute or chronic form. In this study, miR 210 was described as the best biomarker of hypoxia. Transcriptomics, especially miRNAomes, will not only complement them as biomarkers to understand the pathological changes in HIE at the cellular and genetic levels, but also can be potential therapeutic targets. Commercially available pharmacological agents have been studied for their ability to mimic endogenous miRNA or decrease the miRNA expression in vitro and in vivo [23]. A recent report showed the circular RNA expression profiles in the peripheral blood of HIE patients [37]. The various biomarkers of genomics and transcriptomes are summarized in Table 1.

In addition to the miRNA studies, there have been a few systematic studies on the expression profiles of other RNAs in HIE. Several differentially expressed microRNAs with upregulation were identified in addition to the differentially expressed mRNAs, long noncoding RNAs (lncRNAs), and circular RNAs (circRNAs) [38]. A microarray study involving the reverse transcriptase PCR of Gene Ontology (GO) and Kyoto Encyclopedia of Genes and Genomes (KEGG) pathways revealed the differential expression of 456 circRNAs in HIE, with 250 upregulated and 206 downregulated circRNAs in comparison to the controls [37]. Long noncoding RNAs (lncRNAs) play essential roles in the regulation of gene expression. In total, 376 lncRNAs and 126 mRNAs were differentially expressed between the HIE and the non-HIE samples [39]. These aberrantly expressed IncRNAs in the central nervous system may be involved in the pathogenesis and progression of HIE.

## 4. Proteomics

In order to provide the groundwork for an integrated understanding of the biological system underlying the disease, proteomics studies seek to close the knowledge gap between functional proteins and genetic data [40]. An understanding of the genome, together with encoded gene products (proteins), will help to provide clear extrapolations regarding the complex biological processes. The cellular phenotype and the objects of natural selection are determined by the functional regulation of cellular processes and the execution of gene-directed activities by proteins [41]. The goal of proteomics is to decode the genetic sequence information about the structures and roles of proteins. The first quantifiable biomarkers that may be evaluated are often transcript levels of particular genes that code for proteins that can respond to perceived stresses, including hypoxia [42].

**Figure 3:**
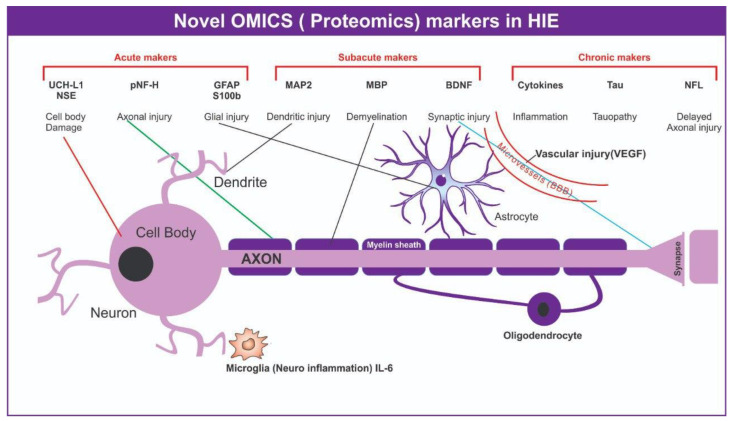
Proteomics markers in neonatal HIE.

Various proteomic candidate markers have been studied for traumatic brain injury (Figure 3). Glial fibrillary acidic protein (GFAP), a cytoskeleton intermediate filament protein of the astrocytes, was found to be a promising marker for HIE [43], with the highest quantity observed 1–2 days after hypoxic injury [44]. The calcium-binding protein S-100B, a major component of glial cell cytosol, was also found to be a biomarker for the diagnosis of HIE. S100B was found in higher concentrations in cord blood samples and the first urine after birth in HIE patients than those samples in controls [16,45]. This protein can be used as a biomarker of HIE if measured immediately after injury [46].

Ubiquitin carboxy-terminal hydrolase L1 (UCHL1) protein, a cysteine protease known as a neuronal-specific protein gene product, has been suggested as a marker for brain injury due to its high and specific expression levels in brain tissue [46,47]. Studies found that the UCHL1 levels were high in the umbilical cord blood of neonates with HIE compared to the samples from controls [10,48]. Activin A, a member of the transforming growth factor β superfamily that plays pivotal roles in the regulation of tissue homeostasis, organ development, inflammation, cell proliferation, and apoptosis [49], has been shown to protect against hypoxic–ischemic damage in animal and cell studies [50]. Different studies have observed increased levels of activin A in the cerebrospinal fluid, urine, and serum of infants with moderate and severe HIE, which can be considered as a biomarker for the early diagnosis of HIE [51]. Tau proteins that stabilize microtubules, specifically cleaved Tau (C-TAU), were also found to be potential biomarkers for HIE. During the process of brain development, Tau proteins aid in signal transduction pathways and interactions with the actin cytoskeleton, neurite outgrowth, and stability [52,53]. In a previous study, asphyxiated newborns had higher levels of the Tau protein in their cord blood than did controls [54]. Additionally, by identifying a large number of endogenous peptides in the cord blood, the total peptidomics analysis reliably detects HIE cases [55].

The resident Immune cells in the brain are called microglia, and the inflammatory reactions to numerous stimuli begin with microglial activation [56]. In both the parenchymal and systemic circulation, cerebral ischemia causes an inflammatory cytokine response. An early and exaggerated increase in pro-inflammatory cytokines and nucleated red blood cells was observed in neonates with HIE [57,58,59]. Various interleukins, such as IL6, IL1b, IL8, TNF-a, and IL10, were found to be upregulated in cord blood and also modulated by therapeutic hypothermia [57,60]. Neuroinflammation is an important feature of brain injury, making cytokines not only potential biomarkers for diagnosis, but also for the prognosis of injury [61]. A neutrophil called chemotaxis factor IL-8, which recruits neutrophils to injured areas, was found to increase in the acute phase of injury in neonates with HIE [47]. Elevated inflammatory cytokines found in newborn blood are associated with ventriculomegaly, severe germinal matrix hemorrhage, and periventricular white matter damage, as determined by ultrasonography [62]. These results indicate that these neuroinflammatory cytokines may be useful as biomarkers for the diagnosis and prognosis of HIE.

During damage, neurons release NSE, an enzyme involved in the brain glycolytic energy metabolism, and its levels are frequently increased. NSE is a highly specific marker for neurons and peripheral neuroendocrine cells [63] and a potential marker for the detection of neuronal cell death due to it high specificity and sensitivity for axonal injury [64]. NSE could also be a potential marker for therapeutic monitoring and for the prediction of long-term outcomes [65]. A positive correlation was observed between the serum levels of NSE and the infarct volume after acute ischemic stroke [66]. Celtik et al. also studied serum NSE as a predictor of HIE severity [67]. The attachment of the cytosolic surfaces of multilayered compact myelin is caused by myelin basic protein (MBP), the most prevalent protein in the central nervous system myelin. MBP can be a specific biomarker of white matter lesions or nerve fiber demyelination, as its concentration increases rapidly in the blood and cerebrospinal fluid with the severity of myelin damage [47]. Neonates with moderate to severe HIE had considerably greater serum MBP levels than those with mild HIE and no injuries [47]. Brain-derived neurotrophic factor (BDNF) was found to be persistently elevated in neonates with HIE [51]. This neurotrophin, secreted by central nervous system neurons and astrocytes, is involved in neuronal survival and synaptic plasticity [46,47]. Liu et al. [68] also showed an increase in serum matrix metalloproteinase-9 (MMP-9) in neonates with HIE. MMP-9 is involved in the disruption of the blood–brain barrier by degrading brain vasculature basement membrane components [47]. An increase in plasma concentrations of vascular endothelial growth factor (VEGF) was observed with an increased severity of neonatal HIE [47]. In hypoxic–ischemic events, the angiogenic factor VEGF, which is released by microglia and astrocytes, is overexpressed. By encouraging vascular endothelial cell growth and angiogenesis, it defends neurons and glial cells [47]. Red blood cell production is significantly influenced by the hormone erythropoietin (EPO), which is produced in the kidneys. It was discovered that in reaction to HIE, it reduced inflammation, attenuated oxygen-free radicals, decreased caspase activation, and increased neurogenesis [2,69,70]. Animal model studies and clinical trials showed that combining hypothermia with EPO improved the recovery of the outcome [71,72]. A recent meta-analysis suggested that erythropoietin as a monotherapy may improve the outcome after HIE in low- and middle-income countries where therapeutic hypothermia is not available. [73]. However, in clinical scenarios, all markers need to be assessed holistically as per the different associated conditions (Table 2).

Hypoxia–ischemia triggers an inflammatory cascade in the brain, amplified by the activation of immune cells and the influx of peripheral immune cells. A novel post-translational modification of the inflammation in neonatal hypoxic–ischemic encephalopathy, lactylation, can be a marker for diagnosing birth asphyxia [79]. The ischemia-modified albumin (IMA) levels might also have a predictive role in the identification and prevention of hypoxic disorders, as they increase in cases of ischemia of the liver, heart, brain, bowel, and kidneys [80].

The identification of a novel biomarker can be done based on the current molecular pathways underlying the onset or course of the disease, or it can be done objectively by identifying proteins that are expressed differently in the two groups under study [81]. Currently, untargeted approaches in biomarker development are favored by MS-based proteomic techniques, which significantly enhance the number of novel biomarker candidates [82,83]. The MS detection of protein biomarkers in the blood is far more likely to emerge as a standard for the evaluation of HIE or the pathophysiological process. In addition, as protein synthesis and metabolism are the primary means through which pathophysiological processes express themselves in the body, the detection of trace amounts of abnormal biomarkers may occur before HIE is clinically apparent. Consequently, this may evolve to a new paradigm, where the early initiation of an intervention might be possible before the pathophysiology emerges clinically with the deterioration of the patient. This approach can limit the mortality and morbidity in the future.

## 5. Metabolomics

Metabolomics is a multi-parametric method for simultaneously identifying and quantifying low molecular weight biochemicals (metabolites) present in biological fluids or tissues in order to track the changes brought on by pathological situations [84]. The metabolic levels and relative ratios reflect the metabolic function, and any out-of-range perturbations are often suggestive of disease. By offering biomarkers for diagnosis and treatment options, metabolomics significantly advances our understanding of pathophysiological–biochemical alterations [85]. This aids in comprehending the final metabolic phenotype of the HIE damage, reflecting the influence and interplay of both the HIE injury received and the patient-specific genomic and transcriptomic inputs. A biochemical signature of HIE metabolomics could be utilized to assess the injury severity in addition to clarifying the disease process (Table 3).

A number of studies on the metabolomics of perinatal asphyxia have highlighted the potential benefits of metabolomics analyses in human neonatal studies. In a targeted metabolomic analysis, Weiss et al. showed a significant increase in 29 of 148 measured metabolites in the umbilical cord blood of infants with either asphyxia or HIE compared to matched healthy controls [97]. Three distinct metabolite classes, i.e., amino acids, acylcarnitines, and phosphatidylcholines, were found to be altered in neonates with HIE. Compared to the controls, higher acylcarnitine levels were found in infants with both asphyxia and HIE, as well as a significant increase in a group of eight amino acids. As observed in two studies, the Acyl-CoA levels may be the earliest and a cardinal irreversible event in ischemia, preceding the formation of oxygen-free radicals and NO [57,98]. The clinical results (neurological examination, brain magnetic resonance imaging (MRI), and electroencephalogram) before discharge and at six months were studied over a 10 year period in 67 HIE patients by López-Suárez et al. [86]. In this work, dried blood spots taken on day 3 for newborn screening were subjected to a tandem-MS analysis to determine the acylcarnitine profiles with lower amounts of long-chain acylcarnitine (Table 3). As blood levels of butyryl carnitine (C4-carnitine) are linked with NSE levels and aberrant MRI images, this appears to be a reliable prognostic marker in HIE. Most of the long-chain acylcarnitine levels are increased and C4 levels are decreased during hypothermia treatment [86]. Paired estimations of acylcarnitines and amino acid metabolites on day 1 and post-treatment can help to improve the predictions of the severity of HIE at the time of birth and the response to treatment, which can limit the future morbidity.

The oxidative stress derived from perinatal asphyxia is linked to neonatal brain damage. Lipid peroxidation biomarkers can estimate oxidative stress in HIE with hypoxia and reperfusion injury. Sánchez-Illana et al. developed and validated a comprehensive liquid chromatography and TMS (liquid chromatography (LC)-MS) approach for the quantification of isoprostanoids in 150 plasma samples collected from newborns, including 20 full-term neonates with HIE [87,99]. Isoprostanes are prostaglandin-like compounds produced by the peroxidation of arachidonic acid caused by free radicals rather than by the enzyme cyclooxygenase (COX). Due to their stability, selectivity, and specificity, they are regarded as the gold standards for the in vivo evaluation of lipid peroxidation [100]. Isofurans (isoFs) and neurofurans (neuroFs) were detected in a preliminary biomarker study in newborns with HIE undergoing therapeutic hypothermia treatment [87,101].

Sarafidis et al. using a targeted metabolic technique (LC-MS) to compare the urine metabolic profile of neonates delivered at or after 36 weeks of gestation with HIE to that of healthy controls [99]. Their findings revealed notable variations in a number of metabolites between neonates with HIE and controls, suggesting that these compounds may one day be used as disease biomarkers. Leucine–isoleucine, phenylalanine, tyrosine, tryptophan, threonine, and aspartate were shown to be lower in the urine on the first day of life compared to in the controls. The neonates with HIE were reported to have considerably lower levels of organic acids including kynurenine and hippuric acid in their urine [88]. It is possible to link tissue hypoxia and the ensuing metabolic acidosis in newborns with HIE directly to lactic acid, a key end product of the fermentation of pyruvate, to yield two ATP molecules during anaerobic metabolism. A single lactate measurement may not provide conclusive information regarding the duration of asphyxia, the fetal response to hypoxia, or the danger of a subsequent encephalopathy. However, the severity of lactacidemia may reflect the degree of fetal hypoxia–ischemia [53]. When evaluated within the first 12 h, they discovered noticeably elevated blood and salivary LDH levels [102].

The urinary metabolite profiles measured using MS and analyzed using bioinformatic methods showed that the concentrations of eight urinary organic acids (ethylmalonate, 3-hydroxy-3-methylglutarate, 2-hydroxy-glutarate and 2-oxo-glutarat, glutarate, methylmalonate, 3-hydroxy-butyrate and orotate) were elevated and significantly associated with the prognosis of a neurodevelopmental handicap with high sensitivity and specificity [90]. The severity of HIE can be predicted based on a model using 3-hydroxybutyrate, glycerol, o-phosphocholine, and succinate [91]. The duration of hypoxia can be ascertained via a plasma metabolic score that combines the signals of three metabolites, namely choline, xanthine, and hypoxanthine, which can be compared to lactate [103]. Hypoxia decreases the blocking effect of the magnesium ion channel, causing a rapid influx of Ca^+2^ into the cell, which has been reported to be the major cause of cell death [92]. According to a previous study, newborns with HIE who had a poor outcome had considerably lower serum ionized calcium concentrations shortly after birth. Low quantities may signify harm to several organs, particularly the brain [104]. Similar to the previous study, another one showed that hypoxia can influence iron metabolism, resulting in a considerable rise in the concentration of lipid peroxidation and the rate at which non-protein-bound iron (NPBI) can be detected in plasma in newborn children with HIE [93]. Specific metabolic patterns in newborns with HIE can be found using focused metabolomic screening.

In summary, in clinical scenarios, assessing a combination of metabolites can lead to finding the severity and prognosis of HIE in newborns (Table 3).

Longini et al. examined the effects of hypoxia on newborn urine metabolites in order to highlight variations in the metabolic profile of healthy newborns compared to asphyxiated neonates using an untargeted metabolomics technique combined with ^1^H nuclear magnetic resonance (NMR) spectroscopy. They demonstrated that the metabolites strongly characterizing the asphyxiated group are lactate, glucose, TMAO, threonine, and 3-hydroxyisovalerate [94]. The benefit of a ^1^H NMR metabolomics urine analysis is connected to the quick, non-invasive, and non-destructive evaluation of the metabolic end products. In order to implement early preventive measures to improve the quality of life for neonates with HIE, this could be effective for monitoring early cellular damage and death after a hypoxic–anoxic shock, as well as for the development of short- and long-term negative effects [84]. An umbilical cord blood study using UPLC-MS detected distinct metabolite alterations in HIE and asphyxia, while survivors in the HIE group showed increased levels of methionine and certain acylcarnitines but reduced levels of ornithine, histidine, and arginine [95,105].

## 6. Multi-Omics Approach for HIE

As a single omics marker might not reveal the true pathology of HIE, a multi-omics approach is suggested. This would help to identify a unique metabolic “fingerprint” to be used in the future for the development of diagnostic (extent of disease) and prognostic (predict future outcome) biomarkers, thereby facilitating personalized medical treatment and allowing a more accurate prediction of important clinical outcomes [106] (Table 4). The hypoxia response markers that are important are MALAT1, HIF1α, and RCTOR. In the presence of oxygen, prolyl hydroxylase (PHD) post-translationally modifies HIF1α, allowing it to interact with E3 ubiquitin ligase, leading to proteasomal degradation. The ubiquitination of HIF1α is prevented by MALAT1 under hypoxic conditions, leading to HIF1α accumulation and nuclear translocation, where it regulates the transcription of hypoxia-induced genes. HIF1α mRNA translation is regulated by the mTOR signaling pathway [4].

A recent publication showed a compilation of all available data in an online database [107]. The authors presented a single-cell sequencing (SCS) database and a bioinformatics predictive analytics model. The outcomes provided a better understanding of the roles, showing great potential for further studies on HIE progression, both clinically and via computational models.

## 7. Omics in HIE: Conclusion and Perspectives

**Figure 4:**
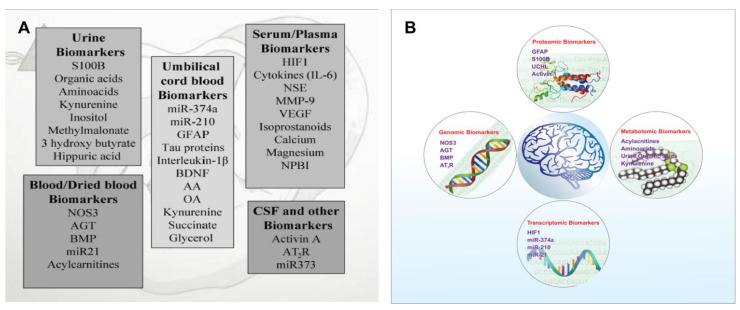
Summary of omics in HIE. (**A**) The biomarkers found in a variety of body fluids, indicating the severity and presence of HIE. (**B**) Biomarkers of HIE as per their biochemistry.

The future of medical research envisions the prospective tracking of health with multiple omics approaches, which could highlight indicators of any disease prior to the actual development of the disease. Furthermore, the applications of omics technologies within a clinical setting can be used in personalized or precision neonatal medicine. At present, only a few cases of omics technologies (genome sequencing or RNA sequencing) have been shown to surpass traditional clinical tests. Therefore, substantial technical and regulatory hurdles exist to incorporating these technologies into clinical practice. However, the integration of these technologies may help to better understand the disease process and may find a place in the future clinical arena. Currently, a few point-of-care devices are approved or in development for biomarker estimation, such as UCH-L1 and GFAP by Abbott (i-STAT Alinity), S100b and NSE by Roche (Cobas), and FFAP and S100B by BioDirection (Tbit). By integrating an automated ELISA, a semi-automated high-sensitivity multiplex workstation, and point-of-care and hand-held devices, we can achieve rapid bedside and specific marker-assessing diagnostics [108]. In addition to the prediction and early diagnosis of HIE, integrative omics is anticipated to become increasingly powerful for disease treatment and prognosis. Information from the transcriptome, proteome, and metabolome, as well as imaging and wearable data, will be used in combination to manage the disease diagnosis, to provide a prognosis, and to guide treatment. In the future, multiple omics measurements (Figure 4) combined with data-driven paradigms will be powerful tools for medical research and will also facilitate clinical diagnoses and treatments. Although many reports use cord blood to diagnose the HIE conditions of neonates [109], a recent report indicated the use of cord blood to treat the conditions successfully, which requires further evaluation of the possible factors in cord blood behind this beneficial role [110]. A combination of the basic molecular mechanisms of neonatal brain injury with advances in neuroimaging might result in the prevention of or reduction in the incidence of HIE-induced lifelong disabilities such as cerebral palsy, epilepsy, and behavioral and learning disorders by undertaking a timely intervention, goal-directed treatment, and early prognostication.

## Figures and Tables

**Table 1:** Various genomics and transcriptomes in HIE.

Genomics					
S. No	Biomarker	Biological Fluid	Analytical Platform	Study Models	Key Findings
1	*NOS3* gene [18]	Blood [19]	PCR and sequencing	Pre-term and full-term neonates with HIE	Strongly suggests an association of the *NOS3* gene polymorphism with the intensity of brain damage.
2	*AGT* gene [20]	Blood [19]	PCR and sequencing	Neonates with HIE	Polymorphism in the AGT gene is associated with HIE.
3	*BMP* gene [21]	Cerebral blood	Neuroanatomical studies in gene knockouts	BMP-3b knockout mouse models	BMP-3b knockout/gene depletion mice exposed to severe hypoxia have a high mortality rate. BMP-3b may play a crucial role for survival in severe neonatal HIE insult.
4	*AT_2_R* gene [22]	RNA extracted from brain tissue	Real-time RT-PCR	Mouse model	Nicotine-exposed pregnant mice showed a vulnerability of brain hypoxic–ischemic injury in neonates. AT_2_R gene repression in the developing brain is linked to perinatal stress and pathophysiological vulnerability of brain HIE.
**Transcriptomics**					
**S. No**	**Biomarker**	**Biological Fluid**	**Analytical Platform**	**Study Models**	**Key Findings**
1	HIF-1 [23]	Serum	qRT-PCR	Neonates with HIE	Expression of miRNA-21 and HIF-1α was found to be higher in HIE infants compared to normal controls.
2	miR-374a [24,25]	Umbilical cord blood	miRNA microarray, real-time PCR	Neonates with HIE	Significant stepwise downregulation of hsa-miR-374a expression in the cord blood of infants with perinatal asphyxia and subsequent HIE.
3	miR-210 and miR-374a [26]	Umbilical cord blood	Real-time PCR	Neonates with HIE	Expression levels of miR-210 and miR-374a were highly different between HIE infants and healthy infants. miR-210 and miR-374a, together with S100B and NSE, can be ideal markers to achieve higher accuracy for the diagnosis of the extent and prognosis of brain injury.
4	miR 210, miR 21, miR 424, miR 199a, miR 20b, and miR 373 [27]	Maternal whole blood	qRT-PCR	Women undergoing induction of labor at term	Approximately 90 microRNAs were induced by hypoxia and modulate the HIF-induced cellular response to hypoxia. miR 210 is the best marker as it is universally induced in response to hypoxia in all cell types and responsible for the adaptation of multiple cellular processes.
5	HIF1A, MALAT1, and RICTOR [4]	Whole-blood RNA	Next-generation sequencing	Neonate with HIE	Striking upregulation in these gene expression profiles at birth in neonatal encephalopathy.
6	RGS1 and SMC4 [4]	Whole-blood RNA	RT-PCR	Neonate with HIE	A total of 855 genes were significantly differentially expressed between the good and adverse outcome groups; among these, *RGS1* and *SMC4* were the most significant. Transcriptomic profiling might be a beneficial tool for rapid risk stratification in neonatal encephalopathy.

**Table 2:** Proteomics markers in HIE.

Proteomics					
S. No	Biomarker	Biological Fluid	Analytical Platform	Study Models	Key Findings
1	GFAP [74]	Umbilical cord blood and neonatal serum	Electro-chemiluminescent sandwich immunoassay	Neonates with HIE	To recognize and keep track of infants receiving cooling therapy who have clinical HIE, GFAP may be used as a biomarker. GFAP is an early biomarker of HIE.
2	S100B [16]	Urine	Immuno- luminometric assay	Neonates with HIE	In term neonates with perinatal hypoxia, elevated S100B protein urine levels appear to indicate a higher risk of neonatal morbidity.
3	UCHL1 [48,75]	Serum	ELISA	Neonates with HIE	In subarachnoid hemorrhage and traumatic brain injury, high levels of serum UCHL1 have been seen, indicating that they are released into the blood after neuronal injury. In neonatal HIE, serum UCHL1 may be investigated as a diagnostic and prognostic marker.
4	Activin A [76]	Cerebrospinal fluid	ELISA	Neonates with HIE	Newborns with severe HIE had higher CSF activin A concentrations than infants without the condition.
5	Tau proteins [54]	Umbilical cord blood	ELISA	Neonates with HIE	The prognosis in HIE can be predicted by serum Tau protein levels on days 3 and 7.
6	Pro-inflammatory cytokines [59]	Serum	ELISA	Newborns with HIE and their mothers	To spot early evidence of brain damage in newborns with prenatal hypoxia, IL-6 in the umbilical cord may be helpful.
7	Interleukin-1β [77]	Umbilical cord blood	Radioimmunoassay	Neonates with HIE	The highest levels of HIE and poor neurological outcomes at 6 and 12 months after birth were best predicted by IL-1. Umbilical IL-1 concentrations can be utilized to predict HIE grades and their negative effects.
8	NSE [67]	Serum	Immunometric assay	Neonates with HIE	NSE is a potential marker for the detection of neuronal cell death due to it high specificity and sensitivity for axonal injury. Serum NSE is an early predictor of HIE severity.
9	MBP [47]	Serum	Chemiluminescent Immunoassay	Neonates with HIE	MBP can be a specific biomarker of white matter lesions or nerve fiber demyelination as its concentration increases rapidly in blood and cerebrospinal fluid with the severity of the myelin damage.
10	BDNF [51]	Umbilical cord blood	ELISA	Neonates with perinatal asphyxia	BDNF levels in cord blood were noticeably greater and were linked with the severity of prenatal hypoxia in neonates. The severity and outcome of neonatal asphyxia could be predicted using BDNF.
11	MMP-9 [47]	Serum	ELISA	Neonates with HIE	Increase in MMP-9 in neonates with HIE. MMP-9 is involved in the breakdown of the blood–brain barrier by degrading brain vasculature basement membrane components.
12	VEGF [47]	Plasma	Chemiluminescent Immunoassay	Neonates with HIE	In hypoxia–ischemia, VEGF, an angiogenic factor released by astrocytes and microglia, is overexpressed.
13	S100A8 [78]	Plasma	LC-MS/MS	Neonates with HIE	The S100A8 levels were significantly increased in all three groups of HIE patients and reflected the severity of HIE.

**Table 3:** Various metabolomics markers in HIE.

Metabolomics					
S. No	Biomarker	Biological Fluid	Analytical Platform	Study Models	Key Findings
1	Amino acids, acylcarnitines, and glycerophospholipids [57]	Umbilical cord blood	LC-MS/MS	Neonates with HIE and asphyxiated infants	Infants with both hypoxia and HIE had higher levels of acylcarnitines compared to controls, but infants with HIE had higher levels of amino acids.
2	Acylcarnitine [86]	Dried blood spots	Tandem-mass spectrometry	Neonates with HIE	Significant increase in C4, C5, C5:1, C6, C6-OH, and C8 acylcarnitine levels and decreased long-chain acylcarnitine levels in HIE neonates. Treatment with hypothermia was linked to decreased C4 levels and elevated levels of the majority of long-chain acylcarnitines.
3	Isoprostanoids [87]	Plasma	LC-MS/MS	Neonates with HIE	Isofurans (isoFs) and neurofurans (neuroFs) detected as preliminary biomarkers in newborns with HIE undergoing therapeutic hypothermia treatment.
4	Organic acids and amino acids [88]	Urine	LC-MS/MS	Neonates with HIE	Amino acids, leucine–isoleucine, phenylalanine, tyrosine, tryptophan threonine, and aspartate were found to be decreased in the urine of HIE neonates compared to controls during the first day of life. Organic acids, such as kynurenine and hippuric acid, were significantly decreased in the urine of neonates with HIE.
5	Kynurenine [89]	Umbilical cord blood	LC-MS/MS	Neonates with HIE	Kynurenine levels in neonates with HIE have significantly decreased.
6	Inositol [88]	Urine	LC-MS/MS	Neonates with HIE	In newborns with HIE, inositol levels increased considerably within the first three days.
7	Urinary organic acids [90]	Urine	LCMS-MS	Newborns with perinatal asphyxia	Eight urinary organic acids (ethylmalonate, 3-hydroxy-3-methylglutarate, 2-hydroxy-glutarate and 2-oxo-glutarat, glutarate, methylmalonate, 3-hydroxy-butyrate, and orotate) in distinct biochemical pathways were elevated in neonates with perinatal asphyxia.
8	Organic metabolites [91]	Umbilical cord blood	H NMR	Newborns with perinatal asphyxia	Infants with severe HIE had significant alterations in acetone, 3-hydroxybutyrate, succinate, and glycerol.
9	Calcium and magnesium [92]	Serum	ICPMS	Neonates with HIE	It was shown that newborns with HIE had lower total serum calcium and magnesium levels. They could be prognostic indicators in HIE.
10	Non-protein-bound iron [93]	Plasma	Photometry	Neonates with HIE	Significant increase in the detectable rate of non-protein-bound iron in the plasma, and the concentration of lipid peroxidation in newborn infants with HIE might be due to the effect of asphyxia on the iron metabolism.
11	Lactate, glucose, trimethylamine-N-oxide, threonine, 3 hydroxyisovalerate, acetate, succinate, citrate, urea, formate [94]	Urine	1H-NMR	Neonates with HIE	Metabolites such as lactate, glucose, trimethylamine-N-oxide (TMAO) with threonine, and 3-hydroxyisovalerate are characterizing markers in the asphyxiated group, with decreases in dimethylglycine, dimethylamine, creatine, succinate, formate, urea, and aconitate.
12	Alanine, valine, phenylalanine, acylcarnitines [95]	Cord dried blood spot	UPLC-MS	Neonates with HIE	The amino acids, alanine, valine, phenylalanine, leucine and methionine; and the acylcarnitines C0, C10, and C2 were elevated in the asphyxiated group, but decreased the citrulline-to-phenylalanine ratio, as well as the levels of histidine and ornithine and the acylcarnitines C14, C16, and C3.
13	Lactose, citrate, galactose, hydroxyl proline, taurine, lysine, oxalates [96]	Urine	^1^H-NMR	Neonates with HIE	An increase in urine metabolites, such as lactose, citrate and hydroxyproline, and a decrease in lactate, taurine, lysine and oxalates.
14	Myoinositol, betaine, acetone, amino acids, sugars, creatine/creatinine ratio, cis-aconitate [84]	Urine	^1^H-NMR	Neonates with HIE	Significant variations between the urine samples from HIE patients and healthy neonates at birth were shown via a multivariate analysis. Myoinositol, betaine, and the creatine/creatinine ratio were increased in HIE infants compared to normal infants.

**Table 4:** Application of multi-omics approaches for predicting the timing and severity of the injury and short-term and long-term outcomes in HIE.

Parameter	Genomics	Transcriptomics	Proteomics	Metabolomics
Timing of injury	*NOS3gene* [18]	HIF-1 [23]	S100B [16] GFAP [74]	Amino acids, acylcarnitines, and glycerophospholipids [57]; urinary organic acids [90]; organic metabolites [91]
Severity of injury	*AT_2_R gene* [22]	miR-374a [24,25]	S100A8 [78], NSE [67], activin A [76]	Acylcarnitine [86]; organic acids and amino acids [88]
Mortality (Short-term outcome)	*BMP* gene [21]	miR-374a [24,25]	NSE [67]	Calcium and magnesium [92]; alanine, valine, phenylalanine, acylcarnitines [95]
Neurodisability (Long-term outcome)	*AGT* gene [20]	miR 210, miR 21, miR 424, miR 199a, miR 20b, and miR 373 [27]; RGS1 and SMC4 [4]	GFAP [74], S100B [16], UCHL1 [48,75], Tau proteins [54]	Amino acids, acylcarnitines, and glycerophospholipids [57]; kynurenine [89]
